# Acute Extrapyramidal Side Effects from Smoked Haloperidol

**DOI:** 10.1155/2021/4177263

**Published:** 2021-10-02

**Authors:** Angeline Pham, Joo-Young Lee, Christopher W. T. Miller

**Affiliations:** Department of Psychiatry, University of Maryland Medical Center, USA

## Abstract

**Introduction:**

Haloperidol is a dopamine receptor antagonist used to treat patients with psychotic disorders. Especially at high doses, haloperidol carries a higher risk of extrapyramidal symptoms (EPS) compared to second-generation antipsychotics. Few cases of haloperidol misuse are found in the medical literature. *Case Presentation*. We describe a patient with schizophrenia who smoked marijuana mixed with crushed haloperidol tablets. After the smoking of cannabis and haloperidol, the patient presented to the emergency department (ED) with suicidal ideation, psychosis, and acute dystonia. With the administration of intramuscular diphenhydramine at the ED, the dystonia resolved in less than an hour. To our knowledge, this is the first report on haloperidol misuse by smoking.

**Conclusion:**

Clinicians should be aware that patients might misuse prescribed antipsychotics via unconventional routes, potentially combined with other substances.

## 1. Introduction

Haloperidol is a butyrophenone antipsychotic that has been widely used in the treatment of patients with psychotic disorders, such as schizophrenia. It is accepted that antagonism of dopamine (D2) receptors mainly accounts for the efficacy of haloperidol in reducing positive symptoms of schizophrenia [1]. High-potency first-generation antipsychotics (FGAs) such as haloperidol, especially when used at high doses and without coadministration of anticholinergics, carry a significant risk of developing extrapyramidal symptoms (EPS) including dystonia, Parkinsonism, akathisia, and tardive dyskinesia [2]. Widespread recreational use of haloperidol or other FGAs has not previously been reported. In contrast, second-generation antipsychotics (SGAs) have been widely misused or abused. This can be explained by the broader pharmacodynamic properties of SGAs, with their sedative and anxiolytic effects that may augment the desired effects of abused substances and limit the associated dysphoria [3].

Few anecdotal reports have been published on the deliberate misuse of haloperidol tablets. One article described an individual who developed catatonia after using haloperidol tablets purchased from the streets to “get high” [4]. Another paper depicted a patient who used haloperidol tablets concurrently with street-made “designer drugs” to counteract their undesirable psychotropic actions and to potentially enhance euphoria; this patient later presented to an emergency department (ED) with torticollis [5]. To our knowledge, there has been no report of the misuse or abuse of haloperidol via smoking or, more generally, inhalation. Here, we describe a patient who initially presented to an ED for psychosis, suicidal ideation, and EPS in the context of smoking marijuana mixed with powdered haloperidol tablets.

## 2. Case Presentation

A 35-year-old African-American male with schizophrenia and a history of several prior inpatient psychiatric admissions presented to the ED for auditory hallucinations, suicidal ideation, and “locked-up muscle” (sic) after smoking cannabis mixed with haloperidol. He reported crushing 30 mg of haloperidol tablets from an old prescription. He smoked the crushed haloperidol with cannabis to “get high” [sic] the day prior to presenting to the ED; however, we were not able to further clarify his motivation behind adding haloperidol to his cannabis. He denied any synthetic cannabinoid or other substance use. He did not report any comorbid medical illness. The patient was provided a wheelchair in the ED due to difficulty with ambulation; he later fell onto the floor while trying to get out of his wheelchair to use the restroom. His vital signs were notable for a blood pressure of 141/95 mmHg, pulse of 84 per minute, respiratory rate of 18 per minute, oxygen saturation of 99% on room air, and temperature of 98.6 F (37.0°C). He refused any lab work on presentation and did not provide urine for a toxicology screening test. Nevertheless, according to numerous medical records between 2013 and 2020, the patient had consistently reported using marijuana but no other substances. At least eight prior urine toxicology results were consistently positive only for cannabinoids. On physical exam, his arms were maintained in a flexed position and were noted to have increased tone bilaterally. Given his stable vital signs, the presentation was more suggestive of acute dystonia than neuroleptic malignant syndrome. The dystonia resolved within 60 minutes after he was given diphenhydramine 25 mg intramuscularly. On reassessment approximately two hours later, he was able to ambulate without difficulty, and upper extremity muscle tone was normal bilaterally. He was transferred to the Psychiatric Emergency Services (PES) for continued treatment. Further investigation revealed that the present ED visit was taking place two days after the patient had been discharged from a month-long inpatient hospitalization for his psychotic symptoms. During these two days, he did not take any psychotropic medications prescribed. During that inpatient stay, he had reported muscle stiffness from another FGA, fluphenazine (he was receiving 5 mg daily by mouth), for which he had been started on benztropine 1 mg twice daily.

After clinical assessment at the PES, he was admitted involuntarily for inpatient psychiatric hospitalization due to his ongoing auditory hallucinations, thought disorganization, and erratic behaviors, consistent with decompensation of his schizophrenia. On the inpatient unit, fluphenazine and benztropine were restarted without recurrence of dystonia. Laboratory test results were obtained five days after his presentation to the ED and showed creatinine phosphokinase of 859 units per liter (U/L) (reference range: 55-170 U/L); otherwise, complete blood count and complete metabolic panels were within normal limits.

## 3. Discussion

This case highlights a previously unreported combination of smoked cannabis with crushed haloperidol tablets, resulting in acute dystonia.

Through D2 receptor blockade in the mesolimbic dopamine pathway, haloperidol helps decrease the positive symptoms of schizophrenia (i.e., delusions, hallucinations, disorganized thinking, and disorganized speech) [1]. However, systematically circulating haloperidol cannot selectively block mesolimbic D2 receptors while sparing other dopaminergic pathways in the brain. When 75-80% or more of D2 receptors in the nigrostriatal dopamine pathway are blocked, EPS is likely to occur [2]. Dystonia, which we presented in our case, is a type of EPS associated with involuntary movements from either intermittent or sustained muscle action. Approximately 25-40% of patients who are treated with FGAs may experience dystonia, with children and young adults being more commonly affected. Acute dystonia typically occurs between 24 and 48 hours after oral administration of an FGA [6].

When antipsychotics block D2 receptors, dopamine is no longer available to suppress acetylcholine release within the nigrostriatal pathway. This leads to acetylcholine overactivity in the basal ganglia, which manifests as EPS [2]. Concomitant use of anticholinergic medications can dampen excess acetylcholine activity resulting from D2 blockade. Therefore, as shown in our case, administration of an anticholinergic (such as diphenhydramine or benztropine) helps reduce EPS from antipsychotics [2]. Studies have shown that intramuscular (IM) benztropine or diphenhydramine administration typically resolves EPS completely within 20-30 minutes [6]. If complete resolution does not occur after the first dose, another one can be given after 30 minutes. In milder cases of dystonia, oral anticholinergics can be used. It should be noted that long-term, concurrent use of an anticholinergic with an FGA is not always indicated because this does not necessarily prevent EPS in someone who has never had this side effect; also, the potential cognitive impact of chronic anticholinergic burden needs to be considered. However, in individuals who *have* experienced EPS induced by an FGA, using an anticholinergic with the FGA may prevent future EPS [7].

Drugs can be misused through many different routes. The nonoral routes allow a higher dose of the drug to quickly reach the brain, bypassing hepatic metabolism and resulting in a more rapid effect, thereby leading to greater reinforcement. There are several studies on intranasal administration of haloperidol but none that investigated the pharmacokinetics of inhaled haloperidol [8]. However, one may speculate on the pharmacokinetics of inhaled haloperidol by extrapolating from those of inhaled loxapine, the only antipsychotic with an inhaled form investigated in controlled studies. Inhaled loxapine is administered through a hand-held, breath-actuated tool, designed to instantly deliver powdered loxapine into the alveoli. Inhaled loxapine exhibits intravenous- (IV-) like pharmacokinetics, with the time to maximum plasma concentration around two minutes, leading to a rapid systemic effect [9]. Acute dystonia can occur within hours of starting oral haloperidol and within minutes if IM or IV routes are used [10]. However, no report has so far documented how quickly acute dystonia can occur after inhaling haloperidol. We cannot infer that inhalation will follow the same pattern as IM or IV routes because it is unclear what percentage of powdered haloperidol reaches systemic circulation.

There is an expansive literature on misuse of antipsychotics, alone or in combination with other substances. The recreational misuse of SGAs has been widely reported, quetiapine being the most common, followed by risperidone and olanzapine [3]. There have been numerous reports of prisoners feigning psychotic symptoms to obtain quetiapine and use its powder intranasally [11]. This popularity of SGAs over FGAs has been explained by the milder side effect profile of SGAs, more specifically a lower propensity to cause EPS. Quetiapine (also called “quell” or “baby heroin”) is well known to be misused together with other psychoactive substances, such as cocaine (a combination known as “Q ball”), marijuana (“Maq ball”), or opioids, as it intensifies the overall sedative/anxiolytic effect and can also mitigate the dysphoria associated with coadministered stimulant intoxication or withdrawal [11]. To date, only one brief report has described smoking cannabis simultaneously with an antipsychotic—in this case, crushed quetiapine tablets [12]. However, this article did not include any clear pharmacologic explication of how this route compares to other forms of administration.

Due to lack of previous research, it is challenging to specify the role of haloperidol when used recreationally in combination with cannabis. Olanzapine, considered an ideal “trip terminator,” has been misused along with other novel psychoactive substances to dampen the severity of undesired psychotic symptoms [13]. Considering the role of olanzapine in polysubstance use, one can speculate that people may use haloperidol along with cannabis to lessen the unpleasant effects resulting from cannabis. A controlled study of coadministration of haloperidol and *Δ*-9-tetrahydrocannabinol (*Δ*-9-THC), the main psychoactive ingredient in cannabis, showed that haloperidol significantly lowered the intensity of psychotic symptoms resulting from *Δ*-9-THC [14]. Furthermore, a drug-drug interaction between cannabis and haloperidol may increase the serum level of haloperidol, potentiating its overall antipsychotic effect as well as the possibility of developing EPS; haloperidol is a substrate of CYP3A4, while *Δ*-9-THC is a possible inhibitor of CYP3A4 [10]. In contrast, smoking tobacco may *lower* the serum level of haloperidol through induction of CYP1A2 (involved in haloperidol breakdown), possibly mitigating EPS [10].

A limitation of our report is that we did not obtain confirmatory testing of haloperidol, haloperidol metabolites, or urine toxicology. However, we reviewed the patient's hospital records to verify that he had been prescribed haloperidol. Additionally, with his reported use of haloperidol and his clinical course—the resolution of dystonia following administration of diphenhydramine—we had a reasonable degree of confidence that the patient did take haloperidol as he reported. Furthermore, with the consistency shown in his medical records about his marijuana use but no other substance, the clinical suspicion was very low that the dystonic reaction may have been caused by an alternative antidopaminergic agent (e.g., contaminants present in substances of abuse). However, such a source of exposure remains a possibility; indeed, this could not have been entirely ruled out even if the clinical team had obtained confirmatory testing detecting haloperidol or its metabolites.

## 4. Conclusion

We presented the case of a patient who inhaled prescribed haloperidol by adding it to his smoked cannabis, leading to an acute dystonic reaction. This case highlights how patients may recreationally use antipsychotics through novel routes, either to potentiate euphoria or to manage the undesirable effects of concurrently used substances. Therefore, prescribing clinicians should be cognizant of the potential for such misuse, particularly in patients who have a known history of substance use.

## Data Availability

The data are not publicly available due to privacy or ethical restrictions.
